# Cardioimmunology of Myocarditis: Targeting the IL-1 Pathway

**DOI:** 10.1007/s11886-026-02390-4

**Published:** 2026-07-03

**Authors:** Emanuele Chiara, Maurizio Pieroni, Michel Obeid, Giacomo Emmi, Danilo Malandrino

**Affiliations:** 1 Department of Medical, Surgical and Health Sciences, University of Trieste, Trieste, Italy; 2 Clinical Medicine, Immunology and Rheumatology Unit, Cattinara University Hospital, Trieste, Italy; 3 Department of Experimental and Clinical Medicine, Florence, Italy; 4 Cardiomyopathy Unit, Careggi University Hospital, Florence, Italy; 5 Centre Hospitalier Universitaire Vaudois, Immunology and Allergy Service, University of Lausanne, Lausanne, Switzerland; 6 Centre for Inflammatory Diseases, Department of Medicine, Monash University, Monash Medical Centre, Melbourne, Australia

**Keywords:** Myocarditis, Interleukin-1, Anakinra, Canakinumab, Rilonacept

## Abstract

**Purpose of Review:**

Myocarditis is a heterogeneous inflammatory syndrome with aetiologies ranging from viral infection and drug hypersensitivity to systemic autoimmune/autoinflammatory disease and immune checkpoint inhibitor (ICI) therapy. In response to these triggers, the innate immune response and inflammasome activation can amplify myocardial injury *via* IL-1, providing a mechanistic rationale for IL-1 pathway inhibition as a targeted therapeutic strategy. This review synthesizes preclinical and clinical evidence for IL-1 blockade in myocarditis and related inflammatory cardiac syndromes.

**Recent findings:**

The immune system plays a central role in the pathogenesis of myocarditis, both in idiopathic/viral cases and in systemic autoimmune and autoinflammatory diseases (SAAD). Interleukin-1 (IL-1) has emerged as a key mediator linking inflammation to myocardial dysfunction, supported by experimental and translational evidence implicating activation of the NLRP3 inflammasome. Clinically, the randomized trial of anakinra in acute myocarditis (ARAMIS) did not improve outcomes in a largely low-risk cohort, but accumulating case reports and small series suggest potential benefit in fulminant/hyperinflammatory myocarditis and chronic active refractory myocarditis. In contrast, IL‑1 inhibitors have robust randomized and real-world evidence in recurrent pericarditis, supporting a myo‑pericardial inflammatory *continuum* and validating IL‑1 pathway engagement as an actionable target in selected inflammatory cardiac phenotypes. Together, these findings support the evolving concept of cardioimmunology.

**Summary:**

Current management of myocarditis remains largely supportive, with limited disease-modifying options. Anti-IL-1 therapies, particularly anakinra, have shown promising efficacy in selected severe and refractory cases, with a favourable safety profile. However, evidence is mainly derived from case reports and small series, and robust randomized data are lacking. Key clinical questions remain unresolved, including patient selection, timing of initiation, and treatment duration. Future studies should focus on identifying inflammatory endotypes and evaluating targeted immunomodulatory strategies, including in emerging settings such as ICI-associated myocarditis in which IL‑1 blockade remains investigational.

## Introduction

Myocarditis is a heterogeneous inflammatory disease of the myocardium, defined by clinical, imaging, and histological criteria according to the latest European Society of Cardiology (ESC) guidelines [1]. Inflammation of myocardium can result from a wide range of triggers, including viral infections, autoimmune disorders, and drug or toxin exposure. However, only a minority of patients with suspected myocarditis undergo endomyocardial biopsy [2], thus limiting mechanistic understanding and precision therapies development. Population-based studies about incidence and prevalence of myocarditis are limited, and most epidemiological studies are restricted to selected groups, likely resulting in an underestimation of the true disease burden. A large Swedish registry reported an incidence of 6.3–8.6 per 100,000 inhabitants, mostly in young men [3], while the current global burden of cardiovascular disease data reports a prevalence of 4.2–8.7 per 100,000 in the age range between 35 and 39 years [4]. Viral infections, particularly enteroviruses and adenoviruses, represent the most common cause in developed countries. However, several non-infectious aetiologies should be recognized, such as autoimmune diseases or immune stimulation (e.g., vaccines or immune checkpoint inhibitors [ICI]). Among these, immune-check point inhibitor (ICI)-associated myocarditis has emerged as a rare but severe immune-mediated form, requiring prompt recognition and early immunosuppressive treatment [5].

Clinical presentation spans a broad spectrum, ranging from chest pain and dyspnoea to life-threatening arrhythmias and fulminant heart failure (HF). Whereas acute viral myocarditis can resolve spontaneously, a dysregulated immune-mediated response, especially in genetically predisposed patients, can lead to chronic inflammation, impaired left ventricular contractility, and clinical progression to chronic HF [1]. Diagnosis relies on a combination of clinical findings, biomarkers (cardiac high-sensitivity troponin, inflammatory markers), cardiac magnetic resonance (CMR) imaging, and, in selected cases, endomyocardial biopsy (EMB). EMB remains necessary in high-risk cases to guide therapy and is recommended (Class IC) in recent guidelines [1]. Treatment is aetiology-dependent, but supportive care still represents the cornerstone of management [1]. While glucocorticoids (GC) and immunosuppressive agents are indicated in autoimmune forms and in post-acute virus-negative inflammatory cardiomyopathy, there is still a lack of consensus regarding the use of such therapies in acute myocarditis (AM). By contrast, ICI-associated myocarditis represents a distinct immune-mediated entity, typically driven by dysregulated T-cell activation and associated with high mortality. In this setting, withdrawal of ICI and prompt initiation of high-dose GC with early escalation to additional immunosuppressive strategies are often required [5].

Given the key involvement of the IL-1 pathway in cardiac inflammation, including upregulation of the NLRP3 inflammasome in acute myocarditis [6, 7], selective inhibitors targeting IL-1 signalling have emerged as promising therapeutic strategies.

Anakinra, canakinumab and rilonacept are the currently available IL-1 inhibitors. Anakinra is a recombinant IL-1 receptor antagonist that competitively inhibits the binding of both IL-1α and IL-1β to the IL-1 receptor, with subcutaneous or intravenous daily administration. Canakinumab is a monoclonal antibody that specifically targets IL-1β; the long half-life allows a 30-day subcutaneous administration. Rilonacept is a chimeric fusion protein that binds both IL-1α, IL-1β, and the natural antagonist *via* the Fc portion of a human IgG1 immunoglobulin, acting as an “IL-1 trap”, with weekly subcutaneous administration.

Beyond anakinra, canakinumab, and rilonacept, other IL-1 inhibitors are currently under investigation, with goflikicept, that acts as an IL-1 trap with subcutaneous administration, being the most promising.

This review aims to describe the aetiology, clinical course, prognosis, and current therapeutic management of myocarditis, focusing on the inflammatory response and exploring the IL-1 pathway as a potential therapeutic target, from preclinical evidence to current experience in clinical practice.

## Aetiology, Clinical Course, and Management of Myocarditis

### Aetiology and Immunopathogenesis

Myocarditis encompasses a heterogeneous group of conditions with infectious and non-infectious aetiologies, many of which converge into immune-mediated myocardial injury. Viral infections represent the most common cause [1], particularly enteroviruses, adenoviruses, parvovirus B19, and selected herpesviruses (Epstein-Barr virus, human herpesvirus 6), as well as influenza and coronaviruses. Less commonly, myocarditis is caused by other infections, mainly bacterial, such as *Borrelia* species (Lyme carditis), parasites such as *Trypanosoma cruzi* (Chagas disease), particularly in specific geographic areas.

Non-infectious aetiologies include SAAD like systemic lupus erythematosus, systemic sclerosis, rheumatoid arthritis (RA), adult-onset Still disease (AOSD), eosinophilic granulomatosis with polyangiitis; isolated cardiac immune-mediated forms (e.g., giant-cell myocarditis, GCM); inflammatory bowel diseases, drug or toxin-related injury, including ICI-associated myocarditis, and chest radiation. ICI-associated myocarditis represents a distinct and severe immune-mediated entity, typically driven by dysregulated T-cell activation and characterized by high mortality despite early immunosuppressive treatment [5, 8].

Despite this aetiological diversity, different triggers frequently converge into shared histopathological patterns, most commonly lymphocytic myocarditis (LM), but also eosinophilic myocarditis (EM), GCM, and disease-specific forms such as cardiac sarcoidosis, with additional distinctive inflammatory features observed in settings such as ICI-associated myocarditis.

These shared patterns reflect common downstream immune-mediated mechanisms of myocardial injury.

Viruses may induce myocardial damage either directly or indirectly, by triggering immune activation, disrupting T-cell tolerance to self-myocardial antigens and leading to both humoral and cell-mediated autoimmunity.

In SAAD, myocardial involvement reflects systemic immune dysregulation. Among these, AOSD is the paradigm of innate immunity-mediated disease, with a central role of neutrophils, monocytes/macrophages, and related cytokines (i.e. IL-1, IL-6, IL-18). AOSD is a systemic inflammatory disorder usually affecting young adults, characterized by spiking fever, arthralgia/arthritis, and a salmon-pink rash. Cardiac involvement is quite frequent but usually limited to isolated pericarditis. However, in a recent case series of patients with cardiac involvement, 18% presented myocarditis [9] often associated with refractory disease. Myocarditis is also described in the context of macrophage activation syndrome, a severe complication of AOSD. Although anti-IL-1 agents are the cornerstone of AOSD treatment, conclusive evidence about their effectiveness in the case of myocardial involvement is still lacking.

ICI-associated myocarditis is characterized by dysregulated T‑cell activity (often CD8+), myocardial inflammation, and frequent overlap with myositis and neuromuscular syndromes; macrophages can contribute to inflammatory amplification and tissue injury [10, 11]. Reported incidence is low but non-trivial (~ 0.3–1.4% in published series/meta-analyses), with early mortality reported as high in severe cases. Presentation often occurs early after ICI initiation (commonly within weeks to 3 months), and arrhythmias/conduction disease may be prominent even when LV function is preserved [10, 11].

**Fig. 1:**
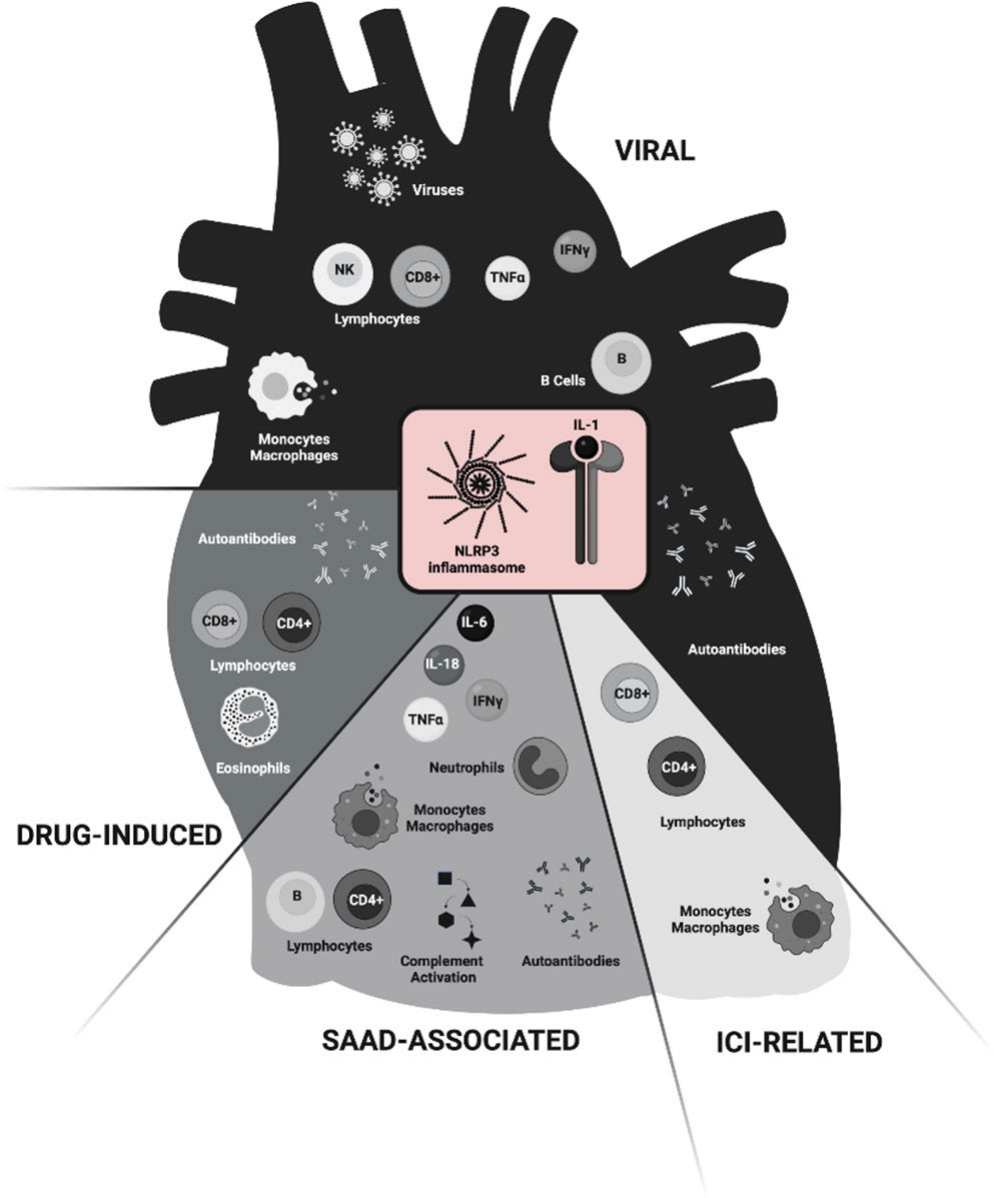
Mechanistic pathways in different aetiologies of myocarditis: a conceptual overview. This figure provides a schematic overview of the immune-mediated mechanisms across different aetiologies of myocarditis, ordered by clinical frequency (four sectors of heart). In viral myocarditis pathogenesis is driven by both direct viral damage and dysregulated immune response (NK cells, monocytes/macrophages, CD8 + cytotoxic T-lymphocytes, TNFα and IFNγ). B cells have a role in production of autoantibodies directed to different cardiac structural proteins. Systemic autoimmune/autoinflammatory diseases-associated myocarditis displays the most heterogeneous pathogenesis, with virtually all cellular types and inflammatory mediators involved, depending on the specific aetiology. Drug-induced myocarditis exhibits a strong eosinophilic infiltrate, with drug-specific autoantibodies and sometimes a hypersensitivity-like reaction. Finally, immune-check point-related myocarditis is specifically marked by a dense infiltration of CD4 + and CD8 + T-lymphocytes. Centrally, the NLRP3 inflammasome and IL-1 pathway represent a common pathogenic axis across all forms, supporting the use of IL-1 inhibitors **Key**: ICI: Immune Checkpoint Inhibitors; IFNγ: interferon-gamma; IL-1: interleukin-1; IL-6: interleukin-6; IL-18: interleukin-18; NK: natural killer; NLRP3: Nucleotide-binding Oligomerization Domain (NOD)-like receptor protein 3; SAAD: Systemic Autoimmune and Autoinflammatory Diseases; TNFα: tumour necrosis factor-alpha

Figure 1 summarizes inflammatory pathways and cellular infiltrates associated with different forms of immune-mediated myocarditis.

### Clinical Course and Prognosis

Myocarditis displays a highly variable clinical course, encompassing acute, subacute, and chronic phases. AM is defined by symptoms lasting ≤ 4 weeks [1] and may be complicated by sustained ventricular arrhythmias, advanced heart block, HF, and cardiogenic shock (fulminant myocarditis). AM may relapse and/or progress to chronic disease when symptoms persist beyond 3 months, possibly leading to inflammatory cardiomyopathy (CMP). Inflammatory CMP is defined by chronic myocarditis associated with cardiac dysfunction and ventricular remodelling, with systolic impairment either associated or not with a dilated phenotype and arrhythmic burden [1]. Inflammatory CMP represents a key step in the progression towards dilated cardiomyopathy (DCM), the late stage of myocardial disease progression, characterized by mostly unreversible damage and HF.

Key determinants of disease resolution versus progression remain incompletely defined. The relative contribution of chronic inflammation, persistent infection, or both in this transition remains to be fully elucidated. Incomplete viral genome-clearing, persistence of cardiac inflammation, and production of autoantibodies supported by a predisposing genetic background are the main mechanisms hypothesised [12]. Interestingly, there is increasing evidence that gene variants coding for structural proteins, particularly desmosomal and sarcomeric proteins, may be present in patients with acute and recurrent myocarditis and affect disease severity [13, 14], showing a certain degree of overlap between gene variants predisposing to myocardial inflammation and inherited cardiomyopathy [15]. The complex interplay between pathogens and host genetic and immunologic background shapes the clinical phenotypes of viral myocarditis (acute, fulminant, chronic, and recurrent), while in SAAD, myocardial involvement usually follows specific inflammatory disease activity.

Prognosis is largely determined by the initial clinical presentation and is aetiology dependent [1]. Low-risk uncomplicated myocarditis (about 75% of unselected cases) typically presents with chest pain with preserved biventricular function, with an overall benign short- and long-term prognosis [16]. Acute complicated myocarditis with arrhythmic presentation, particularly when presenting with HF, has a worse prognosis [17]. Additional negative prognostic factors include fulminant onset, reduced left ventricular ejection fraction (LVEF) at presentation, late gadolinium enhancement in anteroseptal myocardial segments on CMR, distinct autoimmune features (especially in women), and high-titre organ-specific anti-heart autoantibodies and antinuclear autoantibodies [1]. Furthermore, young age and previous history of myocarditis are predictors of relapse. EM, GCM, and cardiac sarcoidosis are associated with a worse prognosis [1]. Major long-term complications include progression to dilated cardiomyopathy and ventricular arrhythmias, including sudden cardiac death [18]. LVEF recovery after myocarditis varies from 50% to 94% depending on the initial presentation [19], and myocarditis is estimated to account for about 10% of dilated cardiomyopathy [20].

Myocarditis is associated with significant mortality, depending on initial presentation and histological subtype. Overall, complicated myocarditis occurs in approximately 4% to 15% of cases, and around 1.2% of patients require durable mechanical circulatory support [21, 22]. The large ESC multicentre myocarditis registry reported 2.7% deaths, 1.7% heart transplant, 0.7% ventricular assistance device implantation, and 3.9% implantable cardiac devices at 1-year follow-up [23–26].

### Current Management and Unmet Needs

Current therapeutic approaches are largely based on ESC recommendations [1]. GC are currently indicated in patients with fulminant forms, or patients with inflammatory CMP refractory to standard HF therapy. Routine immunosuppressive therapy is not recommended in AM with preserved LV function and is reserved for selected conditions (i.e., ICI-induced myocarditis or EM) [1].

Treatment of fulminant cases and prevention of progression to chronic disease remain major unmet clinical needs, reflecting the lack of disease-modifying therapies. Indeed, AM could progress to inflammatory CMP and DCM, irrespective of available treatment strategies [1].

As regards ICI-related myocarditis, a multidisciplinary management involving oncologist is mandatory. Together with ESC recommendations [1], according to specific guidelines from the European Society for Medical Oncology (ESMO) and the American Society of Clinical Oncology (ASCO), management requires immediate ICI cessation, and prompt initiation of high-dose GC with escalation to additional immunomodulatory agents in severe or GC-refractory disease [27, 28]. Emerging strategies in ICI myocarditis prioritize prompt high-dose GC and evaluate escalation to abatacept/JAK inhibition to control T-cell hyperactivity, reflecting a different dominant module than IL‑1 alone [11, 29]. Overall, current ESC guidelines recommend the use of anti-IL-1 drugs for pericarditis, particularly in refractory recurrent cases, and recognise a pathophysiological *continuum* between inflammatory pericardial and myocardial disease, supporting the concept of inflammatory myopericardial syndrome, including perimyocarditis and myopericarditis, according to the leading clinical phenotype.

Table 1 provides a comprehensive overview of pharmacological immune-targeted strategies, bridging evidence-based guideline therapies with novel investigational approaches.

**Table 1:** Immune-targeted therapies for myocarditis and inflammatory cardiomyopathy

Drug	Indication
Immunomodulatory drugs	
Colchicine*	May be considered in myopericarditis to reduce recurrences; not established as routine therapy for isolated myocarditis or inflammatory cardiomyopathy.
IVIG	Third-line therapy in lymphocytic myocarditis (virus-negative); commonly used in pediatric myocarditis.
Glucocorticoids	
Prednisone/methylprednisolone*	Used in fulminant non-infectious myocarditis and in acute myocarditis with impaired LVEF refractory to standard HF therapy; first-line in virus-negative lymphocytic myocarditis, eosinophilic myocarditis, giant-cell myocarditis, cardiac sarcoidosis, and ICI-associated myocarditis; also used in other types of SAAD-associated myocarditis.
Traditional immunosuppressants	
Azathioprine	Selected virus-negative/immune-mediated myocarditis or inflammatory cardiomyopathy, including DCM-like presentations; may also be used in lymphocytic myocarditis, cardiac sarcoidosis and other types of SAAD-associated myocarditis.
Mycophenolate mofetil	Selected immune-mediated myocarditis/inflammatory cardiomyopathy; used in lymphocytic myocarditis; may also be used in cardiac sarcoidosis, other types of SAAD-associated myocarditis and as second-line therapy in steroid-refractory ICI-associated myocarditis.
Cyclosporine	Used in selected SAAD-associated myocarditis and a key component of combined regimens in giant-cell myocarditis; may also be used in lymphocytic myocarditis.
Methotrexate	Selected SAAD-associated myocarditis, preferred second-line steroid-sparing option in cardiac sarcoidosis.
Cyclophosphamide	Reserved for selected severe immune-mediated phenotypes, including refractory cardiac sarcoidosis and selected SAAD-associated myocarditis.
Biologic/targeted therapies	
Abatacept	Second-line option in steroid-refractory ICI-associated myocarditis.
Alemtuzumab	Second-line option in selected refractory ICI-associated myocarditis.
Rituximab	Third-line therapy in selected refractory ICI-associated myocarditis; may also be considered in refractory cardiac sarcoidosis and in selected giant-cell myocarditis/immune-mediated B-cell-driven SAAD-associated myocarditis.
Infliximab	Third-line therapy in selected refractory ICI-associated myocarditis; may also be considered in refractory cardiac sarcoidosis and selected SAAD-associated myocarditis.
Adalimumab	Third-line therapy in selected refractory ICI-associated myocarditis; may also be considered in refractory cardiac sarcoidosis and selected SAAD-associated myocarditis.
Tocilizumab	Investigational/non-established in SARS-CoV-2 related myocarditis, ICI-associated myocarditis, selected SAAD-associated myocarditis (mainly AOSD).
Anakinra*	Investigational/non-established in immune-mediated myocarditis and inflammatory cardiomyopathy; biologically promising, but ARAMIS did not show clear clinical efficacy. Evidence of efficacy is limited to small case series and case reports in viral myocarditis and AOSD-related myocarditis. Currently under investigation in cardiac sarcoidosis.
Canakinumab*	Investigational/non-established in myocarditis and inflammatory cardiomyopathy; human evidence remains anecdotical.
Rilonacept*	Investigational/non-established in myocarditis and inflammatory cardiomyopathy; no human evidence, currently under investigation in cardiac sarcoidosis.
Jak inhibitors	Investigational/non-established in ICI-associated myocarditis.

Indications summarized in this table were adapted from the 2025 ESC Guidelines for the management of pericardial diseases, myocarditis, and inflammatory cardiomyopathy [1] and from literature narrative review

*Indicates inflammasome-targeted therapies. Glucocorticoids indirectly inhibit inflammasome activity by suppressing NF-kB-mediated synthesis of NLRP3 and pro-IL-1β (priming phase, genomic effect)

AOSD: Adult-Onset Still’s Disease; ARAMIS, Anakinra versus placebo for acute myocarditis study; DCM, dilated cardiomyopathy; HF, heart failure; ICI, immune checkpoint inhibitor; IL, interleukin; IVIG: intravenous immunoglobulin; LVEF, left ventricular ejection fraction; SAAD, systemic autoimmune and autoinflammatory diseases

## The Role of Interleukin-1 in Myocarditis: Preclinical Evidence

IL-1 is a key pro-inflammatory cytokine that plays a crucial role in the regulation of immune responses during both acute and chronic inflammation. IL-1 consists of two isoforms, IL-1α and IL-1β, both of which activate immune cells through binding to the IL-1 receptor type 1(IL-1R1). IL-1β production results from the activation of caspase-1 in the NLR family pyrin domain containing 3 (NLRP3) inflammasome in response to a wide range of stimuli, including infectious triggers, such as coxsackievirus B3, a common cause of viral myocarditis [30]. The main cellular sources of IL-1β are monocytes and macrophages, but neutrophils are also involved.

Patients with AM show significant activation of the NLRP3 inflammasome in the heart [31]. Activation of NLRP3 functions as a rapid inducer of an inflammatory response through the production of IL-1β, which is directly involved in cardiomyocyte death, increase arrhythmic risk, decrease of myocardial contractility, cardiac remodelling, and myocardial fibrosis. IL-1 has shown to exert negative inotropic effects in animal and in vitro models. The main mechanisms are inhibition of L-type calcium channels, uncoupling of the β-adrenergic receptor (β-AR) from adenylyl-cyclase [32–38], and inducing transcriptional and posttranslational changes in phospholamban and sarcoplasmic/endoplasmic reticulum (SR) calcium ATPase [39] with consequent impaired contractile function. In various animal preclinical models, IL-1β also showed a causative relationship with arrhythmia development, modifying the normal function of different membrane channels, thus altering calcium and potassium flow [39–45]. Another possible mechanism linked to arrhythmic risk is the decrease of connexin 43 (Cx43), a key protein in cardiac gap junctions involved in electrical coupling and synchronized contraction of the heart [46]. Finally, IL-1β proved to induce cardiac remodelling and fibrosis, leading to DCM and HF in a mouse model [47], and increased susceptibility to arrhythmia in diabetic mice through mitochondrial reactive oxygen species generation that enhances SR Ca2 + leak [48].

The evidence, derived from in vitro and in vivo animal models, found valuable confirmation in humans. The arrhythmic burden was significantly higher in patients with a higher level of IL-1β, both in a cohort of RA patients and of various connective tissue diseases with anti-Ro/SSA positivity (QTc prolongation) [49, 50]. IL-1 levels were also significantly higher in patients with atrial fibrillation (AF) compared with non-AF controls [51].

The concept of inflammatory burden-dependent damage was also suggested in a study by Toldo et al., demonstrating that the degree of inflammasome activation assessed on EMB of patients with acute LM correlates with greater severity of HF at presentation and lower likelihood of functional recovery at 6 months [6].

In addition to IL-1 overproduction, IL-1RA (interleukin-1 receptor antagonist) dysregulation is also thought to be involved in specific types of myocarditis. IL-1RA is the natural antagonist of the IL-1R in the human body that blocks the inflammatory cascade caused by ligand-receptor interaction. Neutralizing antibodies against IL-1RA were observed in young male patients with biopsy-confirmed myocarditis after SARS-CoV-2 mRNA vaccination [52]. These antibodies were associated with a reduction of IL-1RA bioactivity in vitro, low circulating levels of IL-1RA in vivo and were found in patients with evidence of cardiac inflammatory damage [52].

From a therapeutic perspective, blocking IL-1 signalling in experimental models of myocarditis can reduce the recruitment of inflammatory cells to the myocardium, prevent myocardial cell apoptosis, lower the activation of pro-inflammatory pathways, ultimately resulting in improved myocardial healing injury and cardiac function [12]. Treatment with an anti-mouse IL-1β antibody at different stages of enteroviral infection in mice prevented the development of chronic viral myocarditis by reducing inflammation, interstitial fibrosis, and adverse cardiac remodelling [53]. In preclinical animal models of myocarditis, IL-1 blockade with both anakinra and canakinumab, or with targeted plasmid vector, reduced myocardial damage, improved heart function, and decreased markers of systemic inflammation [54–56]. In addition, the IL-1 receptor accessory protein (IL1RAP) blockade with a monoclonal antibody showed reduction of monocytes, T cells, neutrophils, and eosinophils, in the heart in a mouse model of viral myocarditis compared with placebo and anakinra treatment alone [57].

Emerging evidence suggests that similar innate immune pathways may also be involved in ICI-associated myocarditis. In tumor-bearing murine models, NLRP3 inhibition attenuated myocardial inflammation and dysfunction while preserving antitumor efficacy [58]. Consistently, human translational studies have shown that exposure to combinatorial ICI therapy may increase NLRP3 expression and IL-1β secretion in cardiomyocyte-immune cell co-culture systems [59]. In addition, single-cell transcriptomic analyses have demonstrated expansion of monocyte and NK cell populations and activation of pro-inflammatory pathways, including S100A-related signalling, in patients with ICI-associated myocarditis [60]. Although these findings do not directly establish a causal role for IL-1 signalling in humans, they support a mechanistic framework in which innate immune activation and inflammasome pathways may contribute to myocardial injury in this setting.

Taken together, these preclinical data support the role of inflammasome and IL-1 as key mediators of cardiac damage in myocarditis, supporting the rationale for the use of anti-IL-1 therapies in clinical practice.

## IL-1 Inhibition in Myocarditis: Insight Into Clinical Practice

Although in vitro and animal models, alongside limited translational human evidence, support IL-1 as a potential therapeutic target in myocarditis, current clinical evidence is largely derived from case reports and small case series, with few randomized studies available. Table 2 summarizes current literature about anti-IL1 agents in myocarditis.

Anakinra was the most commonly used drug (23/26), mainly in AOSD myocarditis (11/26). Canakinumab was used as first-line therapy in only one case. One patient was treated with rilonacept [82]. Most of the study reported are case reports or small case-series (22/26), with only a limited number of randomized studies available (3/26) [70, 74, 85].

**Table 2:** Summary of case reports and randomized clinical trial of interleukin-1 blockers in myocarditis

First author, year	Article type	Ethiology	IL-1 inhibitor	Other therapies	Outcome
Raffeiner et al., 2010 [61]	Case report	AOSD	Anakinra 100 mg daily sc (12 months)	Pulses of intravenous methylprednisolone, oral prednisone (ineffective)	Complete recovery
Choi A. D. et al., 2014 [62]	Case report	AOSD	Anakinra 100 mg daily sc (12 months)	NSAIDs (ineffective), pulses of methylprednisolone (partially effective)	Complete recovery
Luconi N. et al., 2015 [63]	Case report	AOSD	Anakinra 100 mg daily sc	Pulses of intravenous glucocorticoids plus methotrexate (ineffective)	Complete recovery
Cavalli G. et al., 2016 [64]	Case report	Viral (fulminant)	Anakinra 100 mg daily sc (5 days)	Inotropic agents ECMO, LVAD (ineffective)	Complete recovery
Parisi et al., 2017 [65]	Case report	AOSD (complicated by MAS)	Anakinra 100 mg sc every 6 h (stopped for myelotoxicity); Anakinra 100 mg daily sc	Pulses of intravenous methylprednisolone and intravenous cyclosporine 3 mg/kg/d (increased up to 5 mg/kg) for MAS; IVIG (400 mg/kg/d for 5 days, 2 cycles)	Complete recovery
Cavalli et al., 2017 [54]	Case report	T cells lymphoma complicated by MAS (fulminant myocarditis)	Anakinra 100 mg daily sc	Inotropic agents, IABP (ineffective); CHOEP (after anakinra discontinuation)	Complete recovery
Piel-Julian et al., 2018 [66]	Case report	AOSD	Anakinra 100 mg daily sc	Oral prednisone 1 mg/kg/day, Intravenous methylprednisolone, 1 mg/kg twice a day (ineffective)	Complete recovery
Bello et al., 2020 [67]	Case report	Autoimmune lymphocytic myocarditis	Anakinra 100 mg daily sc	Oral prednisone (1 mg/kg/day); Azathioprine (2 mg/kg/day), recovery of LVEF. Bisoprolol 7.5 mg/day. Amiodarone 200 mg/day (ineffective, symptomatic arrhythmias)	Complete recovery
Abbate et al., 2021 [68]	Case series (2)	Vaccine-associated fulminant myocarditis (Pfizer BNT162b2 mRNA COVID-19 vaccination)	Anakinra 100 mg daily sc	ECMO for both patients	Patient 1: death despite VA-ECMO, (one dose of anakinra administered) Patient 2: complete clinical recovery.
Fiore et al., 2021 [69]	Case report	SARS-CoV-2 myocarditis	Anakinra 100 mg twice daily sc	Hydroxychloroquine 200 mg twice daily (ineffective) Inotropes and IABP (partially effective)	Complete recovery
Kron et al., 2021 [70]	RCT	Cardiac sarcoidosis	Anakinra 100 mg daily sc on top of standard of care versus standard of care only for 28 days		Ongoing
Trpkov et al., 2021 [71]	Case report	SARS-CoV-2 myocarditis	Anakinra 100 mg twice daily sc	Intravenous dexamethasone (one administration)	Complete recovery
Thomson et al., 2021 [72]	Case report	Neisseria meningitidis sepsis complicated by HLH (fulminant myocarditis)	Anakinra 200 mg daily sc	Broad-spectrum antibiotics, vasopressors, CVVHF (ineffective); Intravenous methylprednisolone 1 g daily	Complete recovery
Pina Gonçalves et al., 2022 [73]	Case report	AOSD	Anakinra 100 mg daily sc	Broad-spectrum antibiotics, prednisolone 1 mg/kg/day (ineffective); methotrexate 15 mg/week	Complete recovery from myocarditis, persistent arthritis (switched to tocilizumab and then canakinumab)
Morrow et al., 2023 [74]	RCT	Acute myocarditis	Anakinra 100 mg daily sc within 72 h of hospital admission vs. placebo plus standard of care (max 14 days)	Beta-blockers and ACE inhibitors	No differences in the number of days alive free of any myocarditis complications from randomization to 28 days after hospital discharge
Ono et al., 2023 [75]	Case report	AOSD	Canakinumab 285 mg every 4 weeks sc	Prednisolone 60 mg/day plus tocilizumab 480 mg iv (stopped due to drug-induced liver injury); Prednisolone 120 mg/day and cyclosporin 150 mg/day (ineffective); ECMO, inotropic agents	Complete recovery
Malandrino et al., 2024 [76]	Case series (6 patients)	Chronic idiopathic myocarditis	Anakinra 100 mg daily sc	Failure of conventional cardio-active therapy and/or corticosteroid and immunosuppressants	All patients had an improvement in systemic inflammation, cardiac function and arrhythmic burden
Le et al., 2024 [77]	Case report	AOSD (complicated by MAS)	Anakinra 100 mg every eight hours sc, then 100 mg daily (neutropenia)	Inotropic agents; Hydrocortisone 50 mg every six hours iv; Methotrexate 25 mg subcutaneous (stopped due to neutropenia)	Complete recovery from myocarditis, persistent arthritis (started cyclosporine 100 mg daily and canakinumab subcutaneous)
Thomas et al., 2024 [78]	Case report	SARS-CoV-2 myocarditis	Anakinra (not specified)	Corticosteroids and remdesivir	Improvement of LVEF
Panejiko et al., 2024 [79]	Case report	AOSD	Anakinra 100 mg daily sc	Methylprednisolone pulses (cumulative 3 g) iv followed by oral prednisone 60 mg daily and methotrexate 15 mg per week orally (ineffective)	Complete recovery
Akhila Arya et al., 2024 [80]	Case report	AOSD	Anakinra 100 mg daily sc	Methylprednisolone 1 g iv once a day for three days, followed by oral dexamethasone	Complete recovery
Seminerio et al., 2026 [81]	Case report	AOSD (complicated by MAS)	Anakinra 100 mg daily sc (stopped in the previous 36 h)	Methylprednisolone 500 mg/d for 3 days iv	Complete recovery
Lopez et al., 2025 [82]	Case report	Desmoplakin Cardiomyopathy	Rilonacept 320 mg sc once followed by 160 mg sc/week	Prednisone 40 mg/d, colchicine	Complete recovery
Morini et al., 2026 [83]	Case report	Fulminant myocarditis	Anakinra 100 mg daily sc	Ibuprofen and colchicine, inotropic agents (ineffective)	Complete recovery
Barba et al., 2026 [84]	Case report	Desmoplakin cardiomyopathy	Anakinra (not specified)	ACE inhibitors, beta-blockers, aspirin colchicine, glucocorticoids, and methotrexate (ineffective in preventing recurrences)	Complete recovery
Rosenbaum et al., 2026 (REPAIR-CS, NCT06660732) [85]	RCT	Cardiac sarcoidosis	Rilonacept (320 mg SC on day 1 followed by 160 mg SC weekly) added to standard therapy	Non-biologic standard therapy (corticosteroids and steroid-sparing agents [e.g., azathioprine, methotrexate, mycophenolate, or leflunomide])	Ongoing

ACE: Angiotensin-Converting Enzyme; AOSD: Adult Onset Still Disease; CVVHF: Continuous Venovenous Haemofiltration; ECMO: ExtraCorporeal Membrane Oxygenation; HLH: Hemophagocytic LymphoHistiocytosis; IABP: Intra-Aortic Balloon Pump; IV: intravenously; MAS: Macrophage Activation Syndrome; SC: subcutaneously; RCT: Randomized Clinical Trial

Reported clinical outcomes were generally favourable, with most patients showing improvement after initiation of anti-IL-1 therapy, often with rapid and clinically meaningful responses. However, these observations should be interpreted with caution given the uncontrolled nature of the data and the potential for selection and reporting biases. Anakinra was used in two patients presenting with fulminant hyperinflammatory myocarditis and refractory cardiogenic shock requiring support by extracorporeal membrane oxygenation after COVID-19 vaccine, of which one patient survived [68].

Notably, most patients had acute or fulminant presentations, with only one study evaluating the role of anakinra in chronic myocarditis [76]. Two cases of recurrent myocarditis associated with desmoplakin cardiomyopathy also showed response to anakinra and rilonacept after failure of multiple prior therapies [82, 84]. The ARAMIS trial (Anakinra versus placebo double-blind randomized controlled trial for the treatment of AM; ClinicalTrials.gov identifier: NCT03018834) is the only completed randomized study evaluating the efficacy and safety of IL-1 blockade in AM [74]. A total of 117 patients with CMR-confirmed myocarditis and elevated serum cardiac troponin levels were enrolled and randomized within 72 h of hospitalization to receive anakinra 100 mg subcutaneously once daily (up to 14 days) or placebo. The study did not meet the primary endpoint, showing no significant difference between groups in days alive free of any myocarditis complications at 28 days after discharge [85]. Several limitations constrain the interpretation of these findings. First, the study population mainly included patients with mild disease (predominantly chest pain and ST-segment elevation), with only about 10% of participants presenting with LVEF < 50%. Second, the median duration of anakinra therapy was short (2 days). Finally, the overall event rate was low, with severe complications occurring in only 13.7% of patients, including no HF events and only one ventricular arrhythmia per group. Taken together, these limitations preclude definitive conclusions regarding the potential role of anakinra in myocarditis management, and highlight the importance of appropriate patients’ selection, as well as optimal timing and duration of therapy in future studies. The preliminary results of the MAGiC-ART trial (NCT04017936) provided the first evidence of anakinra efficacy and safety in cardiac sarcoidosis [86], while the ongoing REPAIR-CS trial (NCT06660732) will investigate the efficacy of rilonacept in cardiac sarcoidosis [85].

Overall, these data suggest a potential role for IL-1 inhibitors, particularly in severe forms of AOSD-related myocarditis [61–63, 65, 66, 73, 75, 77, 79–81]. However, selection and survivorship biases of case reports do not allow for conclusive remarks. Regarding other aetiologies, there is still little evidence to support the use of anti-IL-1 therapies with only isolated reports suggesting possible benefit, mainly in severe or refractory cases [54, 64, 67, 69, 71, 72, 75, 78, 83]. Notably, no clinical data are currently available on the use of IL-1 inhibitors in ICI-associated myocarditis, despite emerging mechanistic rationale [58, 87].

Well-designed randomized clinical trials are needed to clarify the role of IL1- inhibition across different form of myocarditis.

## Safety of IL-1 Inhibitors

The safety profile of IL-1 inhibitors is generally favourable, with the most common adverse event being transient localized injection-site reactions. Available evidence supports a favourable safety profile of anti-IL-1 therapies in recurrent pericarditis [88], COVID-19 [89], monogenic and multifactorial autoinflammatory diseases [90] and other cardiovascular diseases like myocardial infarction [91, 92] and heart failure [93]. Among the reported cases, two patients developed clinically relevant neutropenia, both at anakinra dosages higher than the standard dose [65, 77], and one died due to cardiogenic shock despite a single administration of anakinra in the context of severe fulminant myocarditis [68]. Anakinra has been used in critically ill patients with sepsis admitted to the intensive care unit without major safety concern [94] and IL-1 blockade is recommended in AOSD even when bacterial infection is in the differential diagnosis, according to the latest EULAR/PReS international guidelines [95]. Notably, more than half of the patients included in the present review were critically ill and required intensive care. Furthermore, seven patients needed different types of mechanical circulatory support, suggesting that IL-1 inhibitors were used with relative confidence even in severe and potentially life-threatening clinical scenario. Finally, one patient was affected by *Neisseria meningitidis* sepsis [72], highlighting a potentially acceptable risk-benefit profile even in the presence of concomitant bacterial infection.

## IL-1 Inhibitors in Myocarditis: Patient Selection, Monitoring and Tapering

In the absence of high-quality controlled data, any practical guidance on IL-1 blockade in myocarditis should be regarded as mechanistic and investigational rather than prescriptive. Up to date, IL-1 inhibition represents an endotype-targeted rather than a one-size-fits-all strategy: the strongest rationale exists for severe, fulminant, or refractory immune-mediated phenotypes with a prominent systemic inflammatory component, most notably AOSD-related myocarditis, and for the recurrent/refractory idiopathic myocarditis, in the context of inflammatory CMP. Cardiac sarcoidosis and ICI-related myocarditis are emerging settings. Conversely, the neutral result of the ARAMIS trial argues against unselected use in low-risk acute myocarditis with preserved ventricular function and for short duration of therapy [74]. Patient selection should therefore integrate clinical severity, the suspected aetiology/endotype, and the presence of biomarkers of active inflammation. In clinical practice, conventional inflammatory biomarkers (C-reactive protein, erythrocytes sedimentation rates, ferritin and serum amyloid A) are commonly used to phenotype patients and guide therapeutic decisions, together with CMR-defined active inflammation. The same parameters, along with serial troponin, can be used pragmatically to monitor response. Complete blood count is advisable given the risk of neutropenia, particularly at higher-than-standard doses, often required in severe/fulminant myocarditis [65, 77]. A close monitoring of liver parameters and for infections should be also warranted.

With respect to the agent choice, anakinra has been by far the most frequently reported drug and is generally preferred in the acute and critically ill setting because its short half-life allows rapid discontinuation in case of intercurrent infections or adverse events; the standard dose is 100 mg subcutaneously once daily, with higher doses (up to 8 mg/kg/day) described in MAS-associated or refractory cases [65]. Optimal treatment duration and tapering are still undefined: the very short median exposure in ARAMIS (about two days) is widely regarded as insufficient [74], and, translating the evidence from management of pericarditis [1, 96], a more sustained course followed by gradual tapering rather than abrupt withdrawal appears reasonable, although this approach has not been formally tested in myocarditis. Until prospective data will be available, the duration and de-escalation of therapy should be individualised according to aetiology, clinical, laboratory, and imaging response.

## Beyond IL-1: “Inflammasome-targeted” Therapies

IL-1 is not the only potential therapeutic target within the inflammasome pathway, which involves a complex network of interacting molecular components. Emerging findings has expanded interest towards upstream modulation of the inflammasome, particularly targeting NLRP3.

Colchicine a widely used anti-inflammatory agent, inhibits microtubule polymerization, a key process required for NLRP3 inflammasome assembly and activation [97]. Beyond its established role in pericardial disease, emerging data suggest potential benefits in both acute and chronic myocarditis [98–102]. Two ongoing randomized clinical trials (NCT05855746 and NCT06158698) are currently evaluating its efficacy and safety in these settings.

Direct inhibition of NLRP3 has also shown promising results in preclinical models. In a rat model of myosin peptide-induced myocarditis, the NLRP3 inhibitor (MCC950) administered for three weeks, mitigated the leakage of Ca2+, reducing myocarditis-induced arrhythmogenesis and cardiac remodelling [103]. Similarly, dapansutrile, an oral NLRP3 inhibitor, showed improvements in LVEF and exercise time, and reduced IL-1β and IL-18 levels in a phase IB trial on 30 patients with HF [104]. Additional experimental compounds, such as INF200, have shown anti-inflammatory and anti-fibrotic effects, with improvements in cardiac function in preclinical models [105].

In addition to targeted immunomodulatory approaches, some traditional cardiovascular therapies may indirectly exert some anti-inflammatory effects. For instance, sacubitril/valsartan has been shown to inhibit NLRP3 activation, and downstream IL-1β signalling in experimental models of myocarditis [106].

Although still largely exploratory, these approaches reinforce the concept that inflammasome-driven inflammation represents a shared mechanistic pathway across myocarditis and HF, supporting the development of integrated, immunologically targeted therapeutic strategies. Furthermore, focusing on inflammasome pathways as therapeutic targets, could change the “idiopathic” paradigm in myocarditis with an inflammatory phenotype [107], moving from an aetiological classification to a more modern pathogenetic approach.

## Conclusion: Clinical Implications and Future Directions

The IL-1-inflammasome axis provides a compelling mechanistic framework linking innate immune activation to myocardial inflammation and downstream immune responses in myocarditis. However, current clinical evidence supports IL-1 blockade as a potential endotype-targeted strategy rather than a general therapeutic approach for unselected patients.

Anti-IL-1 therapies, particularly anakinra, have shown promising efficacy in selected cases of immune-mediated myocarditis, mainly in severe and refractory presentations, with a generally favourable safety profile, including in critically ill patients. Nevertheless, available evidence remains largely based on case reports and small series, and the only randomized trial conducted to date did not demonstrate a clear benefit in unselected populations. As a result, IL-1 inhibition cannot yet be considered a standard therapeutic strategy in myocarditis.

Several key clinical challenges remain unresolved. Among these, optimal patient selection, timing of treatment initiation, and duration of therapy are critical issues. In viral myocarditis, the optimal timing and selection of patients for immunomodulatory treatment remain uncertain, particularly in balancing modulation of inflammatory pathways with preservation of antiviral immune responses. While IL-1 inhibitors are currently used mainly as rescue therapy in fulminant or refractory cases, earlier intervention in selected inflammatory phenotypes may represent a strategy to modify disease course and prevent progression to inflammatory CMP and DCM.

The identification of inflammatory endotypes represents a major priority for future research. In this context, the development and validation of reliable biomarkers of IL-1 pathway activation, as well as imaging and molecular signatures of disease activity, will be essential to guide patient stratification and treatment decisions.

ICI-associated myocarditis represents a particularly relevant and still largely unexplored setting. Although emerging mechanistic data suggest a role for innate immune activation and inflammasome signalling, current management is primarily based on high-dose GC and T-cell–directed immunosuppression. IL-1 inhibition remains investigational in this context, although it may be biologically plausible in selected overlap phenotypes characterized by prominent systemic inflammation and myopericardial involvement.

More broadly, therapies targeting the inflammasome pathway may offer novel opportunities for intervention across different forms of myocarditis and heart failure. Well-designed randomized clinical trials are needed to clarify the role of IL-1 inhibitors and other inflammasome-targeted therapies, with a focus on enriched populations, mechanistic biomarkers, and imaging-defined endpoints. A multidisciplinary approach integrating cardiology, immunology, and rheumatology expertise will be essential to advance the field of cardioimmunology and improve patient outcomes.

## Key References

- Schulz-Menger J, Collini V, Gröschel J, Adler Y, Brucato A, Christian V, Ferreira VM, Gandjbakhch E, Heidecker B, Kerneis M, Klein AL, Klingel K, Lazaros G, Lorusso R, Nesukay EG, Rahimi K, Ristić AD, Rucinski M, Sade LE, Schaubroeck H, Semb AG, Sinagra G, Thune JJ, Imazio M; ESC Scientific Document Group. 2025 ESC Guidelines for the management of myocarditis and pericarditis. Eur Heart J. 2025 Oct 22;46(40):3952-4041. doi: 10.1093/eurheartj/ehaf192. PMID: 40878297.
  - ⚬ ESC guidelines highlight the role of anti-IL1 agent in pericarditis and support the concept of perimyocarditis and myopericardits as a clinical *continuum*.
- Morrow DA, Verbrugge FH. In-perspective: the ARAMIS double-blind randomized placebo-controlled trial of anakinra for the treatment of acute myocarditis. Eur Heart J Acute Cardiovasc Care. 2023 Sep 25;12(9):627–628. doi: 10.1093/ehjacc/zuad102. PMID: 37648656.
  - ⚬ The first randomized clinical trial assessing anakinra usefulness in myocarditis, proving the absence of clinical benefit, despite in unselected patients.

## Data Availability

No datasets were generated or analysed during the current study.
